# Artificial Intelligence-Assisted Colonoscopy for Colorectal Lesion Detection: Current Evidence, Challenges, and Future Directions

**DOI:** 10.3390/jcm15176558

**Published:** 2026-08-25

**Authors:** Andreas Antzoulas, Francesk Mulita, Vasileios Leivaditis, Elias Liolis, Platon Dimopoulos, Vasiliki Tzelepi, Ioannis Maroulis, Christos-Nikolaos Anagnostopoulos

**Affiliations:** 1 Department of Surgery, University of Patras, 26504 Patras, Greece; 2 Second Department of Surgery, Medical School, Democritus University of Thrace, 68100 Alexandroupolis, Greece; 3 Department of Cardiothoracic and Vascular Surgery, Westpfalz Klinikum, 67655 Kaiserslautern, Germany; vnleivaditis@gmail.com; 4 Department of Oncology, University of Patras, 26504 Patras, Greece; 5 Department of Radiology, University of Patras, 26504 Patras, Greece; 6 Department of Pathology, Patras University Hospital, 26504 Patras, Greece; 7 Department of Cultural Technology and Communications, University of the Aegean, 81100 Mitilini, Greece

**Keywords:** artificial intelligence, computer-aided detection, CADe, colonoscopy, colorectal lesion detection, adenoma detection rate, deep learning

## Abstract

**Background:** Colonoscopy is the gold-standard screening modality for colorectal cancer (CRC) prevention, enabling detection and endoscopic resection of premalignant polyps and reducing CRC incidence and mortality by up to 77% and 53%, respectively. However, colonoscopy effectiveness is substantially dependent on endoscopist expertise, with significant inter-operator variability in adenoma detection rates (ADR) and, consequently, a risk of missed lesions, particularly diminutive and morphologically subtle adenomas. Recent advances in artificial intelligence (AI), specifically computer-aided detection (CADe) and computer-aided diagnosis (CADx) systems utilizing deep learning convolutional neural networks, have emerged as promising technologies to standardize lesion detection accuracy and reduce adenoma miss rates. **Methods:** A focused narrative literature review was conducted examining randomized controlled trials, meta-analyses, and implementation studies evaluating AI-assisted colonoscopy systems across diverse clinical populations and healthcare settings. **Results:** Evidence demonstrates that CADe systems consistently improve ADR, particularly for diminutive polyps and morphologically challenging lesions, though superiority over expert endoscopists remains inconsistent. CADx systems reliably meet ASGE-PIVI performance thresholds for diminutive polyp characterization, supporting implementation of resect-and-discard and diagnose-and-leave strategies. However, substantial heterogeneity exists regarding real-world effectiveness, cost-effectiveness, and optimal implementation frameworks across diverse settings. **Conclusions:** While AI-assisted colonoscopy demonstrates clinical promise in improving lesion detection and enabling optical diagnosis, realizing durable population-level benefit requires the establishment of standardized validation methodologies, large-scale pragmatic trials with patient-centered outcomes, robust regulatory frameworks, and equitable implementation strategies addressing health disparities globally.

## 1. Introduction

Colorectal cancer ranks third in incidence among both sexes and is the second leading cause of cancer-related mortality in the United States [1]. More than 50% of colorectal cancers are linked to modifiable exposures such as smoking, poor diet, excessive alcohol use, sedentary behavior, and obesity, and therefore may be largely preventable [2]. A further proportion of cases and deaths could be averted through appropriate screening and timely access to high-quality treatment [3]. Colonoscopy is the gold standard for CRC screening and prevents colorectal cancer and related mortality by enabling the detection and endoscopic removal of premalignant polyps [4].

Previous colonoscopy exposure has been associated with a substantial reduction in CRC risk, with a population-based case–control study reporting a 77% lower risk of CRC among individuals who had undergone colonoscopy within the preceding 10 years [5,6]. Furthermore, long-term follow-up of the National Polyp Study demonstrated an estimated 53% reduction in CRC mortality following colonoscopic removal of adenomatous polyps over a median follow-up of 15.8 years [7]. However, the effectiveness of colonoscopy is highly dependent on endoscopist technique and examination quality, including bowel preparation and withdrawal time, as well as the adequacy of polypectomy [8]. Colonoscopy performance depends on the operator, since adenoma detection rate (ADR) differs markedly across endoscopists and increases the likelihood of missed adenomas, especially flat or subtle lesions [9,10].

Recent technological advances have introduced artificial intelligence-assisted colonoscopy as a promising modality to improve lesion detection accuracy [11]. Artificial intelligence-assisted colonoscopy, particularly computer-aided detection (CADe) and diagnosis (CADx) systems using convolutional neural networks, has emerged to reduce missed lesions by enhancing visualization and automated polyp detection and characterization [12]. These deep-learning algorithms can accurately localize premalignant lesions and have been developed specifically to improve adenoma detection rate (ADR) during routine colonoscopy [13]. Although AI has been shown in some studies to raise ADR, its principal impact appears to be in lowering interobserver variability and improving detection of diminutive polyps, with less consistent evidence for increased detection of advanced colorectal neoplasia [14].

Our review herein aims to critically examine the technical foundations, summarize current clinical evidence, and evaluate implementation, regulatory, and ethical challenges of artificial intelligence-assisted colonoscopy, with particular emphasis on CADe and CADx systems for colorectal lesion detection and characterization, and to identify priority areas for future research and clinical validation.

## 2. Methods

We performed a focused, non-systematic literature review to identify studies and documents relevant to artificial intelligence-assisted colonoscopy for colorectal lesion detection and characterization. Searches were conducted in PubMed/MEDLINE, Embase, and Scopus from January 2011 through July 2026. The following search string was applied across all databases and adapted to each platform’s syntax: (“artificial intelligence” OR “deep learning” OR “machine learning” OR “convolutional neural network” OR “computer-aided detection” OR “CADe” OR “computer-aided diagnosis” OR “CADx”) AND (“colonoscopy” OR “colorectal polyp” OR “polyp detection” OR “colorectal neoplasm” OR “adenoma detection rate”). Titles and abstracts were independently screened by 3 authors, and potentially relevant articles were retrieved in full text for further evaluation. Any disagreements regarding study eligibility or inclusion were resolved through discussion and consensus among the reviewing authors.

We included clinical studies (randomized controlled trials, prospective and retrospective cohorts), validation studies reporting performance metrics such as adenoma detection rate, sensitivity, specificity, or false-positive rate, and articles addressing implementation, regulatory, ethical, or health-economic aspects of AI-assisted colonoscopy. Review articles and meta-analyses were also included when they provided useful contextual synthesis. We excluded animal studies, isolated case reports, and purely technical or engineering papers that lacked any clinical or validation component, as we aimed to focus on evidence with direct translational relevance to clinical practice. English-language publications were preferentially included; seminal non-English studies were considered when they contributed findings not available in the English-language literature. Reference lists of included articles and relevant reviews were hand-searched to capture additional studies not identified through the electronic search. A total of 104 references were retained for the final narrative synthesis. A formal PRISMA flow diagram was not generated, as this review follows a narrative rather than systematic methodology; however, the search strategy and selection criteria are described above to ensure transparency. For randomized controlled trials evaluating CADe systems and summarized in Table 1, risk of bias was assessed using the Cochrane Risk of Bias 2 (RoB 2) tool. The assessment considered bias arising from the randomization process, deviations from intended interventions, missing outcome data, measurement of outcomes, and selection of the reported results. Domain-level and overall judgments were categorized as low risk of bias, some concerns, or high risk of bias and are summarized in Supplementary Table S1 [15,16,17,18,19,20,21,22,23,24,25,26,27,28,29,30,31,32,33,34,35,36,37,38,39,40,41,42,43,44,45,46,47,48,49,50,51,52,53,54].

## 3. Technical Foundations and Implementation of AI-Assisted Colonoscopy

Gastrointestinal endoscopy provides an ideal setting for developing AI systems capable of assisting endoscopists across multiple aspects of clinical practice [15]. Indeed, AI appears well-suited to enhance quality across virtually all subdomains of endoscopy [16] by standardizing practice and enforcing a minimum quality threshold that is difficult to fall below. Among deep neural network architectures, convolutional neural networks (CNNs) are the most widely employed for medical image analysis [17]. CNNs extract spatial features via stacked convolutional layers. Temporal or spatiotemporal extensions, such as CNN LSTM or 3D CNN, incorporate motion and contextual information from sequential video frames [18]. Recent years have seen the development of computer-aided systems to support optical diagnosis of colorectal polyps. Two complementary types are in clinical use: CADe, which assists endoscopists by detecting candidate lesions, and CADx, which aids in optical characterization and diagnostic classification [19,20]. Selection of algorithms and pipeline design depends on the clinical objectives, the quantity and quality of available annotated data, real-time latency requirements, and the need for robustness across imaging modalities, bowel preparation quality, and endoscope vendors [21].

**Figure 1:**
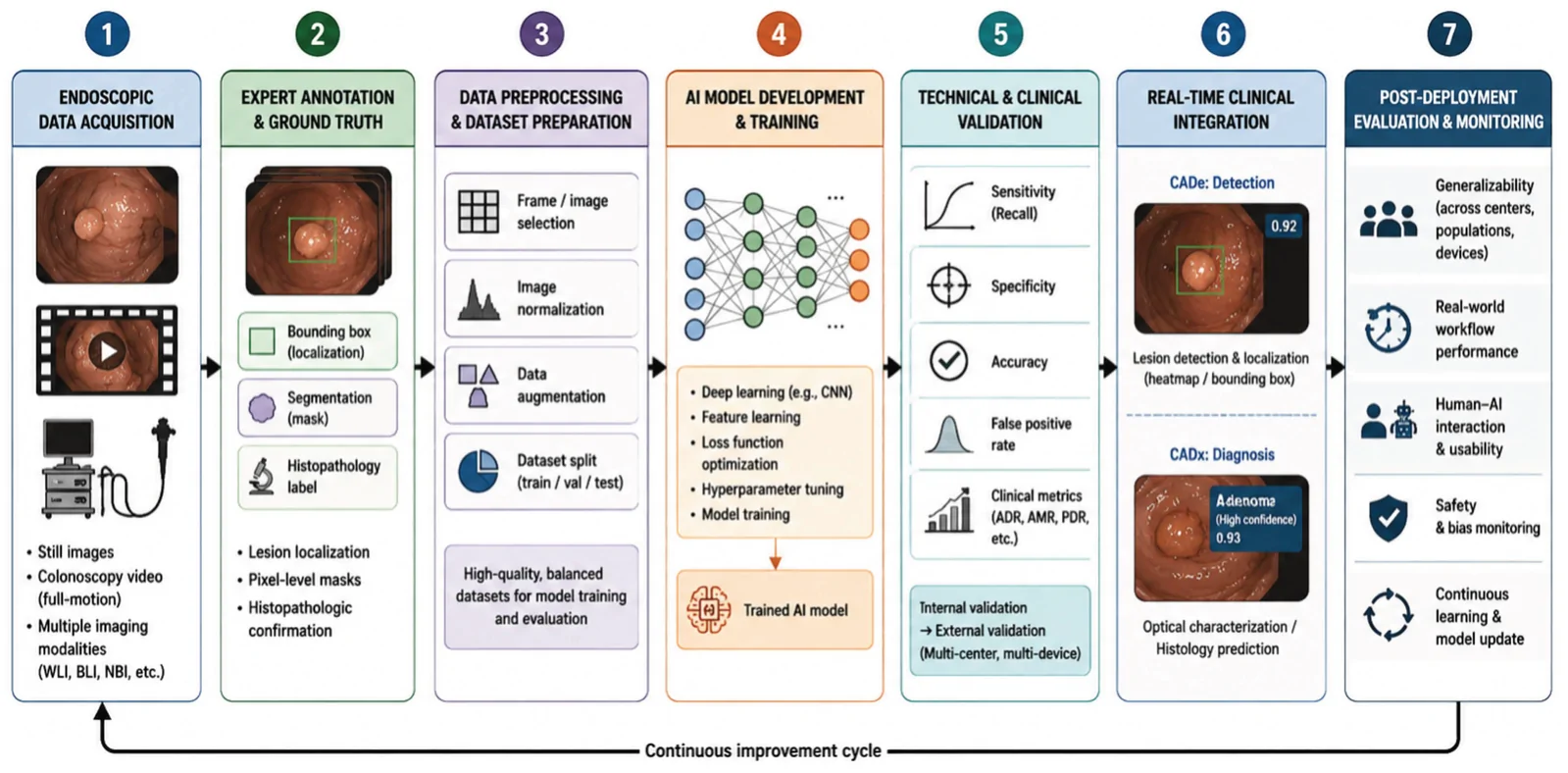
Development and clinical implementation pipeline for artificial intelligence-assisted colonoscopy. The schematic illustrates the principal stages involved in developing artificial intelligence (AI) systems for colonoscopy, beginning with acquisition of endoscopic images and video and expert annotation to establish reference standards. Following preprocessing and dataset preparation, deep-learning models are trained and optimized before undergoing technical and clinical validation. Validated systems may subsequently be integrated into real-time colonoscopy as computer-aided detection (CADe) tools for lesion identification or computer-aided diagnosis (CADx) tools for optical characterization. Post-deployment evaluation and monitoring are required to assess generalizability, clinical performance, human–AI interaction, and sustained effectiveness across different patient populations, endoscopists, imaging systems, and clinical settings. Abbreviations: AI, artificial intelligence; CADe, computer-aided detection; CADx, computer-aided diagnosis; CNN, convolutional neural network.

The implementation of AI in colonoscopy typically follows a structured pipeline that begins with data acquisition. This process can involve capturing still images or full-motion video, where annotated frames and bounding boxes or pixel masks are essential for training AI models [22]. Annotated datasets are crucial, as they provide the ground truth necessary for supervised learning algorithms to discern patterns indicative of pathology. Following data acquisition, preprocessing plays a vital role in enhancing the quality of input data, and techniques such as color normalization, resizing images to a uniform scale, and selecting specific frames from videos are common practices [23]. Data augmentation strategies, such as rotating or flipping images, are employed to artificially expand the training dataset, thereby improving the robustness of the model and mitigating overfitting [24]. In terms of model architectures, various approaches can be utilized. Single-frame CNNs are effective for analyzing individual images, while multi-frame or temporal models, such as CNNs combined with Long Short-Term Memory networks (CNN+LSTM) or 3D-CNNs, are designed to capture temporal dependencies in video sequences [25]. The principal stages involved in the development, validation, and clinical implementation of AI-assisted colonoscopy systems are summarized schematically in Figure 1.

The training and validation of AI models in colonoscopy involve rigorous methodologies to ensure reliability. Cross-validation techniques are frequently employed to assess model performance across different subsets of data, while external test sets provide an unbiased evaluation of model generalization [26]. Transfer learning, where models pre-trained on large datasets are fine-tuned for specific tasks, is often utilized to expedite the training process and improve accuracy, particularly when annotated data is limited [27]. To evaluate the performance of AI models in colonoscopy, several metrics are utilized. Sensitivity and specificity measure the model’s ability to correctly identify true positives and avoid false positives, respectively [28]. Precision and recall provide insights into the model’s predictive accuracy, while the F1 score serves as a harmonic mean of precision and recall, providing a single metric for model performance. Analyses can be conducted on a per-lesion or per-patient basis to derive additional insights into the model’s capabilities [29]. The Adenoma Detection Rate (ADR) is a critical metric in colonoscopy, reflecting the percentage of patients in whom at least one adenoma is detected. False-positive rates, assessed per frame and per procedure, are vital for understanding the clinical implications of AI deployment [30]. Latency and inference speed are also crucial, particularly for real-time applications where immediate feedback is necessary for procedural success [31].

Explainability and uncertainty estimation are increasingly important in the deployment of AI systems in clinical settings. Techniques such as saliency maps and class activation maps provide visual insights into which parts of an image influenced the model’s decision, enhancing clinician trust [32]. Confidence scores can indicate the reliability of model predictions, helping clinicians weigh the AI’s recommendations alongside their own expertise [33].

**Figure 2:**
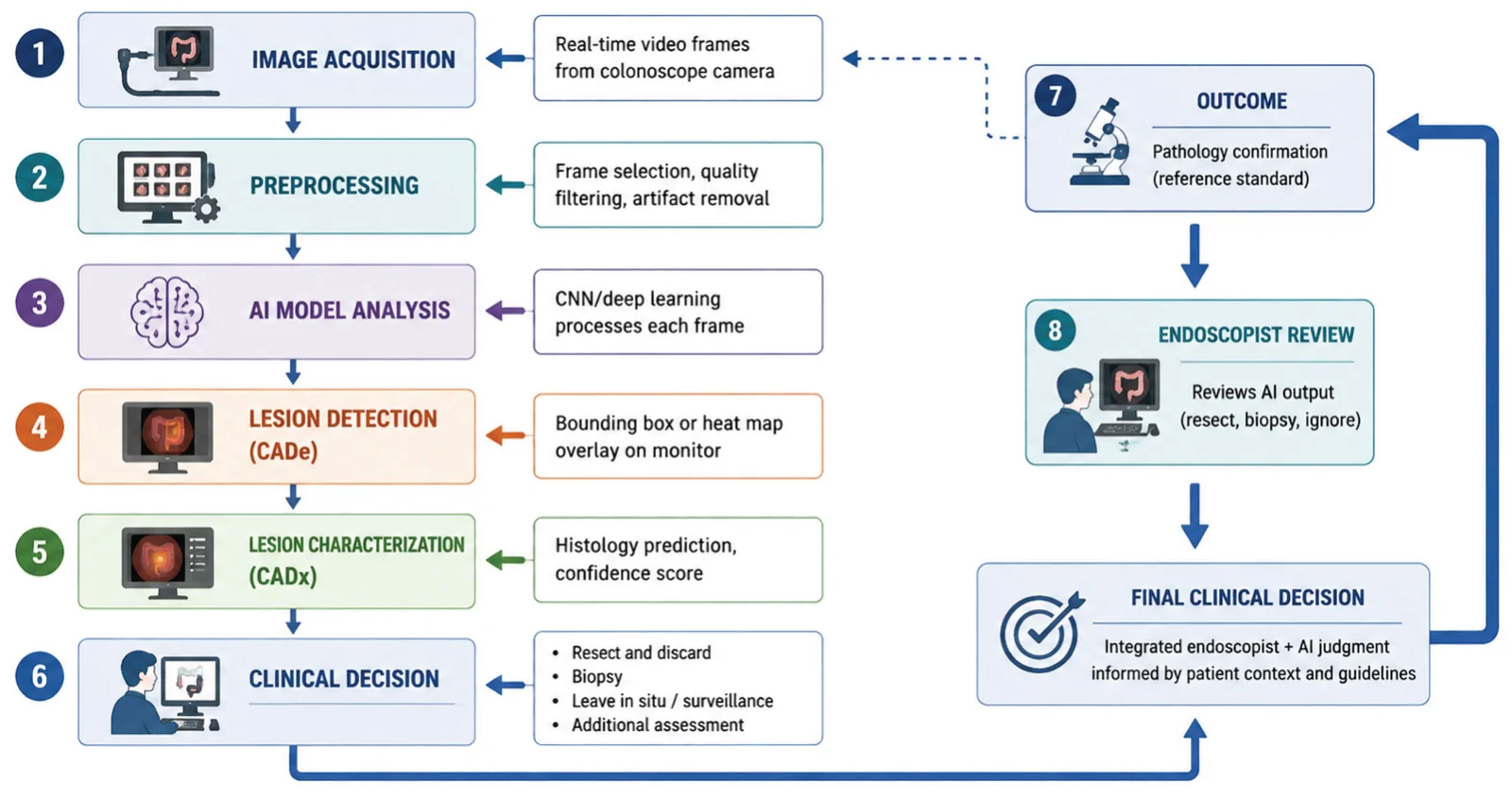
Schematic overview of the artificial intelligence-assisted workflow during colonoscopy.

Regulatory and deployment considerations are paramount when integrating AI into clinical workflows. Decisions regarding on-device versus cloud inference impact latency and data security, with on-device solutions often preferred for real-time applications due to their reduced latency thresholds [34]. Integration with existing endoscopy systems must be seamless to ensure user acceptance and operational efficiency. Compliance with regulatory standards, such as those set forth by the FDA or CE marking in Europe, is crucial for the validation and adoption of AI technologies in clinical practice [35]. The real-time workflow of AI-assisted colonoscopy, from image acquisition and preprocessing through CADe-based lesion detection and CADx-based characterization to endoscopist interpretation, clinical decision-making, and subsequent pathological confirmation, is illustrated in Figure 2.

## 4. Polyp Detection

Computer-aided detection (CADe) systems for colonoscopy represent one of the most extensively studied and clinically advanced applications of artificial intelligence in gastrointestinal endoscopy, driven by the recognition that missed adenomas during screening and surveillance colonoscopy contribute significantly to interval colorectal cancers and suboptimal screening effectiveness [36]. Adenoma miss rates in routine practice range from 20% to 26%, with higher rates for diminutive, flat, right-sided, and sessile serrated lesions, underscoring a persistent quality gap despite established guidelines and quality metrics such as adenoma detection rate (ADR) [37]. CADe systems leverage deep learning—primarily convolutional neural networks trained on large annotated datasets of colonoscopy images and video—to provide real-time, automated alerts when polyps are detected in the endoscopic field of view, functioning as a continuous “second observer” to augment endoscopist vigilance and reduce perceptual errors [38]. Over the past decade, clinical evidence for CADe has matured from proof-of-concept offline validation studies through prospective real-time randomized controlled trials, establishing that CADe assistance can significantly increase ADR, reduce adenoma miss rates, and improve detection of challenging lesion subtypes in diverse clinical settings and across endoscopist experience levels [39].

Clinical evidence from randomized controlled trials evaluating computer-aided detection (CADe) systems in colonoscopy demonstrates variable but generally favorable effects on adenoma detection, with substantial heterogeneity attributable to study design, population characteristics, endoscopist experience, and CADe system performance. Among parallel-design RCTs conducted in screening and mixed-indication populations, several multicenter studies reported statistically significant improvements in adenoma detection rate (ADR): a study [40] demonstrated an absolute ADR increase of +14.4 percentage points (54.8% vs. 40.4%, *p* < 0.05) using the GI Genius system in Italy; another investigation [41] reported a +8.3% improvement (53.6% vs. 45.3%, RR 1.18, *p* < 0.05) in fecal immunochemical test (FIT)-positive patients; and a pragmatic trial [42] showed a +8.1% improvement (42.5% vs. 34.4%, *p* = 0.005) in a community-based U.S. setting. Similarly, research [43] found a +6.0% absolute ADR gain (34% vs. 28%, OR 1.36, *p* = 0.030) in a double-blind Chinese trial, while investigations [44] reported an +11.8% improvement (59.4% vs. 47.6%, *p* = 0.018) using the CAD EYE™ system (FUJIFILM Corporation, Tokyo, Japan) in Japan.

In contrast, several studies failed to demonstrate statistically significant ADR improvements: an analysis [45] found borderline increases in polyp detection rate (PDR) but non-significant ADR gains (71.4% vs. 65.0%, *p* = 0.09) among high-performing U.K. screening endoscopists; a study [46] reported no ADR difference (35.9% vs. 37.2%, *p* = 0.774) in U.S. community practice using EndoVigilant (Millersville, MD, USA); and a multicenter trial [47] observed nearly identical ADRs (38.4% vs. 37.7%, *p* = 0.43) in a European-Canadian setting, attributing the null finding to unexpectedly high baseline control-group performance. Tandem-colonoscopy studies consistently demonstrated CADe’s capacity to reduce adenoma miss rates: investigations [48] reported a reduction in miss rate from 40.0% to 13.89% (*p* < 0.0001); research [49] showed a decrease from 36.7% to 13.8% (*p* < 0.0001); and a trial [50] found a reduction from 31.25% to 20.12% (OR 1.80, *p* = 0.0247), whereas analyses [51] did not observe a statistically significant reduction in proximal adenoma miss rate (20.0% vs. 14.0%, *p* = 0.07) despite improved detection rates.

Secondary outcomes revealed that CADe consistently increased adenomas per colonoscopy (APC) in multiple trials [40,41,44,52], enhanced detection of diminutive and flat lesions, and, in some cases, improved sessile serrated lesion (SSL) detection [39,45], although several studies reported concomitant increases in non-neoplastic resections, raising concerns about specificity [46]. Four-arm trials [53] demonstrated synergistic benefits when combining CADe with computer-aided quality improvement (CAQ) systems for withdrawal monitoring (COMBO ADR 30.60% vs. CADe alone 21.27%, *p* = 0.024).

Narrative synthesis of the available trials suggests that CADe efficacy may be modulated by baseline endoscopist ADR, with greater absolute gains observed among lower-performing operators, and by procedural context, with screening and surveillance populations showing more consistent benefits than diagnostic cohorts [40,42,44,46]. Given the heterogeneity in trial design, clinical populations, procedural indications, CADe platforms, baseline endoscopist performance, and reported detection endpoints, no de novo quantitative pooling of effect estimates was undertaken in the present narrative review.

The risk-of-bias assessment of the randomized evidence is summarized in Supplementary Table S1. Overall, none of the included trials was judged to be at high risk of bias; however, most were classified as having some concerns, predominantly because of the inherent difficulty of blinding endoscopists to real-time CADe assistance. Awareness of active AI support may influence inspection behavior, withdrawal technique, or operator vigilance and could therefore contribute to variation in observed effect estimates. The CADe-DB trial by Wang et al. [43] was judged to be at low overall risk of bias, reflecting its double-blind design and use of a sham system. Concerns across the remaining RoB 2 domains were generally limited, particularly where randomization procedures were adequately described, outcome data were largely complete, and adenoma detection was histopathologically confirmed. Accordingly, differences in methodological quality may account for part of the heterogeneity in reported CADe effects and should be considered alongside baseline endoscopist performance, study population, procedural indication, outcome definition, and CADe platform when interpreting the magnitude and generalizability of the observed benefits [54].

Collectively, these RCTs support the clinical utility of CADe as an adjunct to colorectal cancer screening and surveillance colonoscopy, though variable performance across settings underlines the need for ongoing optimization, standardized evaluation frameworks, and operator training to maximize real-world effectiveness.

The cost-effectiveness of implementing computer-aided detection (CADe)-assisted colonoscopy in screening programs remains subject to considerable debate. Short-term economic analyses demonstrate increased healthcare expenditure due to heightened adenoma detection necessitating expanded pathological services and potentially shortened surveillance intervals. The following economic evidence is presented as illustrative rather than as a comprehensive health-economic review, given the narrative scope of the present manuscript. A pooled meta-analysis by Mori et al. [55] revealed that the proportion of patients requiring more intensive surveillance according to U.S. guidelines increased from 8.4% (control) to 11.3% (CADe group; RR 1.35, 95% CI 1.16–1.57), thereby imposing substantial pressure on healthcare systems. Conversely, long-term economic modeling by Areia et al. [56] utilizing microsimulation methodology demonstrated that CADe implementation in a U.S. population cohort could prevent approximately 7194 colorectal cancer cases and 2089 related deaths annually, with net cost savings of USD 290 million. This paradigm is supported by the World Endoscopy Organization position statement on artificial intelligence in colonoscopy [57], which acknowledges that while CADe increases short-term healthcare costs through enhanced adenoma detection, these incremental expenditures may be offset by downstream cost reductions associated with prevention of advanced colorectal cancer and its treatment. Thus, the economic burden of CADe implementation must be weighed against potential population-level cancer prevention benefits and long-term healthcare savings. Additional research quantifying the cost-effectiveness ratio across diverse healthcare systems and populations is warranted to inform clinical and policy-level decision-making regarding CADe adoption and integration into screening programs [58]. Accordingly, although available modeling studies suggest that the long-term prevention of CRC may partially or completely offset the initial costs associated with CADe implementation, their conclusions remain dependent on healthcare-system-specific assumptions regarding acquisition costs, reimbursement, surveillance intensity, adenoma-to-carcinoma progression, and downstream cancer-treatment costs.

Computer-aided detection systems have demonstrated efficacy in enhancing adenoma detection, particularly for lesion subtypes conventionally associated with higher miss rates due to perceptual limitations, including diminutive polyps, non-polypoid morphologies, right-sided adenomas, and sessile serrated lesions [59]. Although real-world effectiveness studies have yielded variable results, retrospective analyses are subject to inherent methodological biases that may confound outcome estimates. Notably, CAD-assisted colonoscopy enables average-performing endoscopists to achieve adenoma detection rates comparable to expert high-detector operators, thereby standardizing diagnostic quality across diverse clinical settings [60]. This performance-leveling capability is particularly significant given the exponential increase in colonoscopy demand associated with population-based screening implementation, which precludes universal access to specialized referral centers. Consequently, CADe systems have the potential to address healthcare disparities and promote equitable quality of care despite resource constraints [61]. This objective aligns with the European Society of Gastrointestinal Endoscopy position statement, which emphasizes that artificial intelligence in endoscopy should prioritize elevating diagnostic competency among less-experienced practitioners rather than further optimizing performance among already-accomplished operators [62]. In this framework, CADe demonstrates meaningful clinical utility by narrowing the performance gap between practitioners of varying skill levels and facilitating standardized, high-quality adenoma detection across heterogeneous healthcare systems [63].

## 5. CADx for Polyp Characterization

Computer-aided diagnosis (CADx) systems represent the second major application of artificial intelligence in colonoscopy, focusing on optical characterization of detected polyps to predict histology in real time and inform clinical decision-making [64]. While expert interventional endoscopists employing advanced mucosal imaging techniques can achieve high diagnostic accuracy for polyp characterization, this capability requires specialized training, extensive experience, and procedural time that are often unavailable in routine community endoscopy settings [65]. Accurate optical histology prediction is of particular clinical importance in two common scenarios: first, for diminutive polyps (≤5 mm), where reliable characterization enables implementation of cost-saving “resect-and-discard” and “diagnose-and-leave” strategies; and second, for larger polyps, where histologic prediction guides appropriate referral pathways for advanced endoscopic or surgical resection [66].

Multiple image-based studies and three systematic meta-analyses have demonstrated that CADx outperforms non-expert endoscopists in overall histology prediction; however, these same meta-analyses have consistently shown that CADx does not surpass the diagnostic accuracy of expert endoscopists [67,68,69]. Furthermore, real-time colonoscopy studies have failed to demonstrate that CADx significantly improves sensitivity or specificity of histology prediction when used by endoscopists of varying experience levels: Barua et al. found no significant difference in sensitivity (90.4% vs. 88.4%) or specificity (85.9% vs. 83.1%) when comparing CADx-assisted non-expert endoscopists with standard practice [70], while Li et al. reported that CADx was inferior to expert endoscopists in both sensitivity (61.8% vs. 70.3%, *p* < 0.001) and overall accuracy (71.6% vs. 75.2%, *p* = 0.023) [19].

Despite this variability in overall performance, CADx has demonstrated robust and clinically meaningful accuracy in the specific context of diminutive polyp characterization, which has important implications for reducing pathology workload and healthcare costs [71]. The American Society for Gastrointestinal Endoscopy (ASGE) Preservation and Incorporation of Valuable Endoscopic Innovations (PIVI) initiative established performance thresholds for optical diagnosis technologies to support two strategies aimed at reducing the histopathological burden of diminutive colorectal polyps: the “resect-and-discard” strategy, which permits resection of diminutive non-rectosigmoid polyps without pathologic analysis provided that optical diagnosis achieves >90% agreement with pathology-based surveillance interval recommendations; and the “diagnose-and-leave” strategy, which allows diminutive rectosigmoid hyperplastic-appearing polyps to be left in situ provided optical diagnosis achieves a negative predictive value (NPV) >90% for adenomatous histology [72]. CADx systems have comprehensively met and exceeded these PIVI thresholds: multiple image-based studies report NPVs of 96–97% and sensitivities of 92.3–98.1% for diminutive polyp characterization when CADx is used by non-expert endoscopists [71,73].

Although CADx accuracy remains comparable to, rather than superior to, expert endoscopists in this setting, widespread adoption of CADx would enable endoscopists of all experience levels to safely implement resect-and-discard and diagnose-and-leave strategies, thereby improving the cost-effectiveness and efficiency of population-based colorectal cancer screening programs [74]. Rondonotti et al. evaluated CADx in real-time colonoscopy for patients with at least one diminutive rectosigmoid polyp and demonstrated that AI-assisted high-confidence predictions were achieved in 92.3% of polyps, with an NPV of 91% and 97.4% agreement with European Society of Gastrointestinal Endoscopy (ESGE) surveillance interval guidelines; notably, while initial AI-assisted accuracy was significantly higher among expert (91.9%) versus non-expert (82.3%) endoscopists, a significant learning-curve effect was observed such that, for the final 50 polyps, NPV was equivalent between non-experts (95.2%) and experts (93.9%) [75]. The principal clinical and validation studies evaluating CADx for colorectal polyp characterization, together with their diagnostic-performance outcomes and potential clinical implications, are summarized in Table 2.

Computer-aided diagnosis (CADx) systems demonstrate high accuracy in differentiating neoplastic from non-neoplastic polyps and identifying invasive cancers. While these systems do not consistently outperform expert endoscopists, their principal value lies in elevating the diagnostic capability of average-performing endoscopists toward expert level. This enables safe implementation of conservative management strategies for diminutive polyps (resect-and-discard, diagnose-and-leave) and appropriate referral guidance for larger polyps requiring advanced resection. By standardizing diagnostic accuracy across diverse settings, CADx systems reduce inter-operator variability and promote evidence-based adenoma management regardless of endoscopist experience level.

## 6. Overview of Commercially Available AI Platforms

A handful of AI-assisted colonoscopy platforms have cleared regulatory hurdles and made their way into prospective clinical trials. They vary quite a bit under the hood—from their basic system architecture and specific functions to the datasets they were trained on and the depth of their clinical evidence. Table 3 breaks down how these major commercial systems stack up against one another, helping set the stage for the research priorities we outline next.

## 7. Future Research Priorities

Advancing the clinical translation and widespread implementation of artificial intelligence systems in colonoscopy requires systematic addressing of methodological rigor, technical robustness, regulatory innovation, and health equity considerations through coordinated interdisciplinary research initiatives [76]. The field currently lacks consensus regarding minimum dataset specifications, annotation protocols, performance reporting standards, and benchmark methodologies, which impede reproducible research, transparent cross-system comparison, and evidence synthesis [77]. Establishment of internationally harmonized guidelines specifying image resolution standards, minimum lesion numbers stratified by histology and morphology, annotator qualification criteria, and comprehensive histopathologic linkage protocols is essential [78]. Development of open-access benchmark datasets and standardized challenge platforms modeled after established paradigms in medical imaging informatics would facilitate objective algorithm comparison, identify persistent performance gaps, and accelerate iterative improvement of AI systems [79].

Large-scale, multicenter, pragmatic randomized controlled trials with prospective enrollment and long-term follow-up are required to move beyond intermediate endpoints such as adenoma detection and characterization accuracy and rigorously evaluate patient-centered outcomes, including interval colorectal cancer incidence, cancer-free survival, quality of life, and comprehensive health-economic metrics [80]. Such trials must encompass diverse endoscopist experience levels, healthcare settings including both academic and community centers, and heterogeneous patient populations to establish external validity and inform reimbursement and implementation policies.

Substantial research is needed to address critical technical limitations, including the development of domain adaptation techniques enabling reliable performance across endoscope platforms, imaging modalities such as white-light, narrow-band, and chromoendoscopy, and institutional practices [81]. Investigation of continual learning algorithms that incorporate new clinical data while preventing performance degradation or algorithmic drift is essential, as is systematic evaluation of model behavior on rare morphologies and edge cases, including diminutive flat lesions, sessile serrated lesions, and early cancers [82]. Multi-site validation demonstrating consistent performance across diverse hardware, software, and procedural environments remains a priority.

Next-generation systems integrating clinical metadata, including age, family history, and genetic predisposition, procedural variables such as withdrawal time and bowel preparation quality, wide-field imaging characteristics, and downstream histopathology findings, have the potential to enable risk-stratified, personalized adenoma management and individualized surveillance interval assignment [83]. Such multimodal approaches may optimize resource utilization and tailor interventions to patient-specific risk profiles, though rigorous prospective validation is required.

Systematic human-factors research is essential to characterize optimal human–machine interaction paradigms, including investigation of alert design parameters such as temporal frequency, visual salience, and acoustic signals that maximize diagnostic benefit while minimizing alarm desensitization and clinician fatigue [84]. Calibration of clinician trust and reliance on AI outputs, identification of clinical scenarios where AI recommendations should supersede or support human judgment, and development of evidence-based training protocols will inform interface design and operational procedures that preserve appropriate human oversight while leveraging AI capabilities [85].

Current regulatory pathways assume static algorithms; however, deployed AI systems require adaptation to new data and evolving clinical contexts [86]. Establishment of governance frameworks for certifying and longitudinally monitoring continuously learning systems is imperative, including prospective post-market surveillance registries, federated learning architectures preserving data privacy, standardized metrics for detecting algorithmic drift, and protocols for system revalidation after substantial updates [87]. Ongoing research systematically characterizing which morphologic features, imaging patterns, and clinical contexts favor AI performance versus human expertise and where algorithms struggle will refine algorithm design and identify optimal complementary roles for human and machine intelligence in clinical practice [88].

In aggregate, addressing these research priorities through collaborative, interdisciplinary scientific endeavor will establish the rigorous evidence base, technical standards, and implementation frameworks necessary for responsible, equitable, and clinically effective global adoption of artificial intelligence in colonoscopy.

## 8. Health-Economic Considerations and Global Implementation

While most research on AI-assisted colonoscopy focuses on accuracy, the financial reality of rolling this technology out deserves just as much attention [89]. AI brings real added costs—hardware upgrades, software licensing, ongoing maintenance, and staff training [90]. These expenses have to be balanced against potential future savings like catching more adenomas early, reducing interval cancers, and avoiding repeat procedures [30]. Right now, reliable cost-effectiveness data is hard to come by. Although a growing number of health-economic modeling studies have evaluated AI-assisted colonoscopy, the available evidence remains limited and highly dependent on model assumptions and healthcare-system-specific inputs [91]. On top of that, insurance and reimbursement models in most health systems haven’t caught up to AI-driven care, leaving hospitals and clinics in the dark about whether adopting these tools makes financial sense [92].

These economic barriers are even more pressing in low- and middle-income countries (LMICs). Colorectal cancer rates are climbing in these regions, yet endoscopic capacity, trained specialists, and digital infrastructure are already stretched thin [93]. Most current AI models were built and tested in high-resource academic hubs across East Asia, Europe, and North America [94]. They may not hold up well when run on older endoscopes, in settings where bowel preparation quality varies widely, or in patient populations with different disease patterns and lesion types [95]. Closing this gap will mean developing lightweight, hardware-agnostic AI tools, training models on locally representative data, and designing economic frameworks tailored to the financial realities of LMIC health systems. Without a deliberate push for equal access, AI-assisted colonoscopy risks deepening the global divide in cancer outcomes rather than closing it.

## 9. Methodological Quality of the Included Literature

Although the available evidence generally supports a beneficial role for AI-assisted colonoscopy in improving adenoma detection, the strength of this evidence is tempered by several methodological limitations. Most pivotal RCTs were conducted at high-volume academic centers with experienced endoscopists, raising concerns about selection bias and limiting generalizability to community practice where baseline adenoma detection rates and operator expertise may differ substantially [96]. Blinding of the endoscopist is inherently difficult in AI-overlay trials, as awareness of real-time AI assistance may alter inspection behavior and withdrawal technique, introducing performance bias; notably, only the study by Wang et al. [43] employed a double-blind design. Furthermore, while most trials were powered for adenoma detection rate as the primary endpoint, clinically important secondary outcomes such as advanced adenoma detection, sessile serrated lesion identification, and false-positive rates were frequently underpowered, and no trial to date has reported long-term outcomes such as interval colorectal cancer incidence or cancer-related mortality [97].

The interpretation of published meta-analyses is further complicated by marked heterogeneity across studies in terms of the AI systems evaluated, study populations (screening vs. surveillance; Western vs. East Asian cohorts), outcome definitions (per-polyp vs. per-patient analysis), and comparator conditions, as reflected by high I^2^ values reported in several pooled analyses [98,99,100]. Several randomized trials involved direct commercial funding, manufacturer-provided equipment or software, or investigator relationships with AI-system manufacturers, although the nature and extent of industry involvement varied substantially across studies (Supplementary Table S2). Such relationships do not themselves establish bias but should be considered when interpreting the evidence and underscore the importance of independent external validation.

Taken together, these considerations suggest that while the current evidence base is promising, it should be interpreted with caution, and future research should prioritize multicenter pragmatic trials in community settings, standardized outcome reporting, and long-term follow-up to bridge the gap between improved detection and demonstrated clinical benefit.

## 10. Limitations

Several limitations of this review deserve consideration when interpreting its findings. Because this work was structured as a narrative rather than a systematic review, it did not follow a prospectively registered systematic-review protocol, which leaves some potential for selection and reporting bias [101,102]. Although a structured RoB 2 assessment was performed for the randomized controlled trials summarized in Table 1, this assessment was limited to the randomized evidence and was not extended to all study designs included in the broader narrative synthesis. Restricting our search to English-language publications may also have resulted in the omission of relevant evidence from non-English-language research.

Beyond these methodological constraints, the AI technology itself presents several real-world challenges. Most current systems operate as “black boxes,” delivering outputs without fully revealing their underlying decision-making processes, which may make it difficult for endoscopists to develop appropriately calibrated trust or recognize algorithmic errors [103]. Medico-legal liability also remains an area of considerable uncertainty, with no universally established framework defining the respective responsibilities of the physician, healthcare institution, and software developer when AI contributes to a missed lesion or an unnecessary procedure [104]. Compounding this issue is dataset bias: most models are trained on relatively narrow patient populations and specific hardware in high-resource academic centers, potentially limiting performance when deployed in different clinical settings without robust external validation [40,98].

Finally, integrating these tools into networked hospital environments introduces cybersecurity risks, including vulnerability to adversarial inputs, alongside complex requirements for privacy and data protection under frameworks such as the General Data Protection Regulation (GDPR) and Health Insurance Portability and Accountability Act (HIPAA) [105,106]. Addressing these overlapping challenges through targeted interdisciplinary research will be essential before AI-assisted colonoscopy can be considered suitable for widespread and responsible adoption.

## 11. Conclusions

Artificial intelligence-assisted colonoscopy has considerable potential to improve the detection and characterization of colorectal lesions, with CADe systems increasing adenoma detection and reducing missed lesions and CADx systems supporting more consistent optical diagnosis. However, variability in performance across clinical settings, limited evidence regarding long-term patient outcomes, and unresolved economic, regulatory, and implementation challenges currently preclude assumptions of universal clinical benefit. Future research should prioritize robust multicenter validation, standardized evaluation, long-term patient-centered outcomes, and equitable real-world implementation. AI should therefore be regarded as an adjunct to endoscopist expertise, with its ultimate clinical value depending on the safe and responsible translation of improved diagnostic performance into meaningful benefits for colorectal cancer prevention.

## Figures and Tables

**Table 1:** Summary of Randomized Controlled Trials evaluating Computer-Aided Detection (CADe) systems in colonoscopy for adenoma and polyp detection.

Study (Author, Year)	Country	Design	Population	Sample Size (CADe/Control)	CADe System	Setting	Primary Outcome Metric and Result (CADe vs. Control)	Absolute Difference (%)	RR or OR (95% CI)	*p* Value
Repici 2020 [40]	Italy	Parallel RCT	Screening, post-polypectomy surveillance, FIT+, symptomatic	343/342	GI Genius (Medtronic)	Multi-center (3 sites)	54.8 vs. 40.4	+14.4	RR 1.30 (1.14–1.45)	<0.05 ^‡^
Wang 2020 [48]	China	Tandem RCT	Diagnostic, screening, surveillance; age 18–75	184/185	Not specified (deep learning CADe)	Single-center	13.89% vs. 40.00% (miss rate, not ADR)	−26.11	Not reported	<0.0001
Wang 2020 [43]	China	Double-blind parallel RCT	Diagnostic and screening colonoscopy; age 18–75	484/478	Deep learning CADe system (not named)	Single-center	34% vs. 28%	+6.0	OR 1.36 (1.03–1.79)	0.030
Kamba 2021 [49]	Japan	Back-to-back tandem RCT	Screening and surveillance; age 40–80	179/179	Original deep learning CADe	Multi-center (4 sites)	AMR: 13.8% vs. 36.7% (miss rate, not ADR ^†^	−22.9	Not reported	<0.0001
Ahmad 2023 [45]	United Kingdom (England)	Prospective parallel RCT	NHS Bowel Cancer Screening Program	308/306 (ITT); 298/281 (per-protocol)	GI Genius system	Single-center 8 screening-accredited colonoscopists	PDR (ITT): 85.7% vs. 79.7%; ADR (ITT): 71.4% vs. 65.0% ^‡^	PDR: +6.0%; ADR: +6.4%	Not reported	PDR: 0.05; ADR: 0.09 (NS)
Yao 2022 [53]	China	Prospective, four-group parallel RCT	Not specified	268 (CADe alone)/269 (CAQ alone)/268 (COMBO)/271 (Control)	CADe + CAQ	Single-center	CADe: 21.27% vs. Control: 14.76%; COMBO: 30.60%; CAQ: 24.54%	CADe vs. Control: +6.51%; COMBO vs. Control: +15.84%	CADe vs. Control: NR; COMBO vs. CADe: OR 1.284 (1.033–1.596); COMBO vs. CAQ: OR 1.309 (0.857–2.000)	CADe vs. Control: NR ^†^; COMBO vs. CADe: 0.024; COMBO vs. CAQ: 0.213 (NS)
Shaukat 2022 [52]	United States	Prospective parallel RCT	Screening and surveillance	682/677	CADe device	Multi-center, 22 US board-certified gastroenterologists	47.8% vs. 43.9%	+3.9%	Not reported	APC: *p* = 0.002; ADR: *p* = 0.065 (NS)
Glissen Brown 2022 [50]	United States	Prospective, multi-center, single-blind, randomized tandem RCT	Colorectal cancer screening and surveillance	116/116 (randomized); 111 ^†^/112 ^†^ (analyzed after exclusions)	EndoScreener	Multi-center	AMR: 20.12% vs. 31.25% (34/169 vs. 45/144 adenomas) ^‡^	−11.13%	OR 1.8048 (1.0780–3.0217) for HDWL miss vs. CADe	*p* = 0.0247
Rondonotti 2022 [41]	Italy	Multicenter, randomized parallel RCT	FIT-positive individuals, age 50–74 years, baseline endoscopist ADR >25%	405/395 (total 800)	Real-time deep learning CADe system	Multi-center	53.6% vs. 45.3%	+8.3%	RR 1.18 (1.03–1.36)	*p* < 0.05 *
Lui 2023 [51]	Hong Kong, Vietnam, Singapore	Prospective, multicenter, randomized, tandem-colonoscopy RCT	Consecutive patients undergoing colonoscopy (screening/diagnostic); median age 63 years, 48.6% men	108/108 (analyzed after exclusions; 223 enrolled, 7 excluded)	Computer-assisted detection (CADe) system	Multi-center	Proximal Adenoma Miss Rate: 20.0% vs. 14.0% (not significant); Proximal Adenoma Detection Rate: 44.7% vs. 34.6%	Miss rate: +6.0 (NS); Detection: +10.1	Not reported for miss rate	Miss rate: *p* = 0.07 (NS); Detection: *p* = 0.04
Nakashima 2023 [44]	Japan	Prospective, single-center, randomized parallel RCT	Consecutive colonoscopy patients; mean age 55 years, 72% male	207/208 (total 415)	CAD EYE™ (FUJIFILM Co.)	Single-center	59.4% vs. 47.6%	+11.8	Not reported	*p* = 0.018
Wei 2023 [46]	United States	Prospective, multicenter, randomized parallel RCT	Community-based, non-academic colonoscopy patients	387/382 (total 769)	EndoVigilant	Multi-center (4 community-based endoscopy centers, US)	APC: 0.73 vs. 0.67 (NS) 35.9% vs. 37.2%	™1.3%	Not reported	APC: *p* = 0.496; ADR: *p* = 0.774 (both NS)
Thiruvengadam 2024 [42]	United States	Single-center pragmatic parallel RCT	CRC screening, surveillance, FIT+, diagnostic; median age 55.5 years, 61% female, 72.7% Hispanic	550/550 (total 1100)	GI Genius (Medtronic)	Single-center, community-based (community gastroenterologists)	42.5% vs. 34.4%	+8.1	Not reported	*p* = 0.005
Maas 2024 [47]	Europe and Canada (multicenter: 7 hospitals)	Prospective, multicenter, randomized parallel RCT	Diagnostic, non-iFOBT screening, surveillance; experienced endoscopists	250/247 (total 497 analyzed; 581 enrolled)	CADe DISCOVERY system (PENTAX Medical, Tokyo, Japan)	Multi-center (7 hospitals: university and non-university, Europe & Canada)	38.4% vs. 37.7%	+0.7	Not reported	*p* = 0.43 (NS)
Jeon 2026 [54]	South Korea	Prospective, multicenter, randomized parallel RCT	Screening, surveillance, diagnostic colonoscopy	497/501 (total 998 analyzed; 1004 enrolled)	ENAD-DET-01 (ENdoscopy as AI-powered Device)	Multi-center (2 tertiary centers)	52.3% vs. 36.1%	+16.2	RR 1.940 (1.505–2.499); OR 1.914 (1.467–2.496)	*p* < 0.001

*Abbreviations:* ADR, adenoma detection rate; AMR, adenoma miss rate; APC, adenomas per colonoscopy; CADe, computer-aided detection; CAQ, computer-aided quality improvement; CI, confidence interval; FIT, fecal immunochemical test; NS, not sufficient; OR, odds ratio; PDR, polyp detection rate; RCT, randomized controlled trial; RR, relative risk. *Symbols:* * statistically significant difference as reported in the original study; ^†^ outcome not reported or not applicable; ^‡^ significance reported in the original study without an exact *p* value in the summarized data. *Note:* Outcome measures differed across trials. Parallel-group studies predominantly reported ADR or PDR, whereas tandem-colonoscopy studies primarily evaluated AMR. Accordingly, effect estimates should be interpreted according to the outcome metric specified for each study and should not be considered directly interchangeable.

**Table 2:** Summary of key clinical and validation studies evaluating computer-aided diagnosis (CADx) systems for colorectal polyp characterization.

Study	Study Design/Setting	CADx Approach/Imaging	Population/Lesions	Comparator	Main Diagnostic Outcomes	Principal Findings	Clinical Relevance
Li et al. [19]	Comparative evaluation of AI and human experts	AI-based polyp detection and classification	Colorectal polyps evaluated for histologic classification	Expert endoscopists	Sensitivity; overall diagnostic accuracy	CADx sensitivity was 61.8% vs. 70.3% for expert endoscopists (*p* < 0.001); overall accuracy was 71.6% vs. 75.2% (*p* = 0.023)	Demonstrated that CADx performance may approach but does not necessarily exceed expert optical diagnosis
Li et al. [67]	Comparative study of AI and human experts	AI-based detection and classification of colonic polyps	Colorectal polyp images	Human experts	Detection and classification performance	AI demonstrated high diagnostic performance and was particularly competitive with non-expert assessment, although expert performance remained an important benchmark	Supports the potential of CADx to reduce inter-operator variability rather than replace expert interpretation
Meng et al. [68]	Development and validation study	White-light endoscopy-based CADx	Conventional colorectal adenomas, including lesions with high-grade dysplasia	Histopathology/endoscopic assessment	Diagnostic accuracy for prediction of high-grade dysplasia	CADx demonstrated feasibility for identifying advanced histologic features from white-light endoscopic images	Extends CADx beyond simple neoplastic/non-neoplastic classification toward prediction of clinically relevant histologic severity
Hossain et al. [69]	External video/image-based validation study	Novel CADx using white-light imaging (WLI) and image-enhanced endoscopy (IEE)	115 polyp images from 66 patients; specific analyses of diminutive polyps	Endoscopists; histopathology reference standard	Sensitivity; NPV; accuracy; surveillance-interval agreement	For diminutive polyps, CADx sensitivity was 90.9% with WLI and 95.8% with IEE; NPV was 80.0% and 90.9%, respectively; concordance between histology- and CADx-based surveillance intervals was 93.0% with WLI and 96.0% with IEE	Demonstrated potential to satisfy clinically relevant optical-diagnosis requirements, particularly with image-enhanced endoscopy
Barua et al. [70]	Prospective real-time clinical study	Real-time AI-based optical diagnosis	Colorectal polyps encountered during routine colonoscopy	CADx-assisted vs. unassisted endoscopist assessment; histopathology reference standard	Sensitivity; specificity	Among non-expert endoscopists, CADx assistance did not significantly improve sensitivity (90.4% vs. 88.4%) or specificity (85.9% vs. 83.1%) compared with standard optical diagnosis	Shows that strong standalone CADx performance does not necessarily translate into incremental benefit when incorporated into real-time human decision-making
Yoshida et al. [73]	Clinical evaluation study	CAD EYE; lesion recognition and characterization	Colorectal polyps evaluated in clinical practice	Histopathology/endoscopist assessment	Sensitivity; NPV; diagnostic performance	CAD EYE demonstrated high performance for diminutive polyp characterization, contributing to reported sensitivities of approximately 92.3–98.1% and NPVs of approximately 96–97% across relevant image-based CADx evaluations	Supports use of CADx for optical diagnosis of diminutive lesions and potential implementation of pathology-sparing strategies
van der Zander et al. [74]	Prospective image-based validation and reader-comparison study	CADx using high-definition white-light and blue-light imaging	60 colorectal polyps assessed by 6 expert and 13 novice endoscopists	Expert and novice optical diagnosis; histopathology reference standard	Diagnostic accuracy	CADx accuracy was 88.3% with HDWL and 86.7% with BLI; performance was comparable with expert optical diagnosis and superior to intuitive assessment by less-experienced endoscopists in relevant comparisons	Supports CADx as an equalizing tool capable of bringing non-expert diagnostic performance closer to expert level
Rondonotti et al. [75]	Prospective real-time clinical study (ABC study)	AI-assisted BLI characterization	Patients with ≥1 diminutive rectosigmoid polyp	Expert and non-expert endoscopists; histopathology reference standard	High-confidence diagnosis; NPV; surveillance-interval agreement; learning effect	High-confidence AI-assisted predictions were obtained in 92.3% of polyps; NPV was 91% and agreement with ESGE surveillance intervals was 97.4%. Initial accuracy was higher among experts (91.9% vs. 82.3%), but for the final 50 polyps NPV was similar in non-experts (95.2%) and experts (93.9%)	Provides direct clinical support for CADx-assisted resect-and-discard strategies and suggests that CADx may narrow expertise-related performance gaps

*Abbreviations:* AI, artificial intelligence; BLI, blue-light imaging; CADx, computer-aided diagnosis; ESGE, European Society of Gastrointestinal Endoscopy; HDWL, high-definition white-light imaging; IEE, image-enhanced endoscopy; NPV, negative predictive value; PIVI, Preservation and Incorporation of Valuable Endoscopic Innovations; WLI, white-light imaging. *Note:* Diagnostic-performance measures are not directly interchangeable across studies because of differences in study design, lesion selection, imaging modality, CADx platform, operator experience, confidence thresholds, and definition of the target histologic category. Histopathology served as the reference standard in the principal diagnostic-accuracy studies. The ASGE PIVI framework defines clinically relevant performance thresholds for pathology-sparing strategies, including ≥90% agreement in surveillance-interval assignment for a resect-and-discard strategy and ≥90% NPV for adenomatous histology when applying a diagnose-and-leave strategy to diminutive rectosigmoid lesions [70]. The Hossain external-validation study, for example, specifically reports sensitivity of 90.9%/95.8%, NPV of 80.0%/90.9%, and surveillance concordance of 93.02%/96% for WLI/IEE, respectively. The van der Zander study reports CADx accuracy of 88.3% with HDWL and 86.7% with BLI. These details make the table substantially more informative than simply repeating the narrative conclusions.

**Table 3:** Summary of major commercially available AI-assisted colonoscopy platforms.

System	Algorithm	Validation Status	Clinical Application
GI Genius (Medtronic)	Deep learning CNN	Multicenter RCT	Screening, surveillance
EndoScreener (Wuhan EndoAngel)	Deep learning CNN (YOLO-based)	Multiple RCTs	Screening, quality monitoring
CAD EYE (Fujifilm)	CNN with texture/color analysis	Prospective studies, meta-analyses	Screening, surveillance, optical diagnosis
DISCOVERY (Pentax Medical)	Deep learning CNN	Multicenter RCT	Screening
System	Algorithm	Validation status	Clinical application
GI Genius (Medtronic)	Deep learning CNN	Multicenter RCT	Screening, surveillance
EndoScreener (Wuhan EndoAngel)	Deep learning CNN (YOLO-based)	Multiple RCTs	Screening, quality monitoring
CAD EYE (Fujifilm)	CNN with texture/color analysis	Prospective studies, meta-analyses	Screening, surveillance, optical diagnosis
DISCOVERY (Pentax Medical)	Deep learning CNN	Multicenter RCT	Screening

## Data Availability

No new data were created or analyzed in this study. Data sharing is not applicable to this article.

## Supplementary Materials

The following supporting information can be downloaded at: https://www.mdpi.com/article/10.3390/jcm15176558/s1, Table S1: Risk-of-bias assessment of randomized controlled trials evaluating computer-aided detection (CADe) systems during colonoscopy using the Cochrane Risk of Bias 2 (RoB 2) framework. Table S2: Funding sources, material support, and author-disclosed conflicts of interest in randomized controlled trials of computer-aided detection (CADe)-assisted colonoscopy summarized in Table 1.
