# Supplementary material for: Artificial Intelligence-Assisted Colonoscopy for Colorectal Lesion Detection: Current Evidence, Challenges, and Future Directions
Source: J Clin Med. 2026 Aug 25;15(17):6558. doi: 10.3390/jcm15176558 (PMC13566908; doi:10.3390/jcm15176558)
Supplement: Supplementary file 1 [file jcm-15-06558-s001.zip › jcm-4473545-supplementary.pdf]

Table S1. Risk-of-bias assessment of randomized controlled trials evaluating computer-aided detection (CADe) systems during colonoscopy using the Cochrane Risk of Bias 2 (RoB 2) framework.

Study	D1: Randomization process	D2: Deviations from intended interventions	D3: Missing outcome data	D4: Measurement of outcome	D5: Selection of reported result	Overall judgment
Repici et al., 2020 [40]	Low	Some concerns	Low	Low	Low	Some concerns
Rondonotti et al., 2022 [41]	Low	Some concerns	Low	Low	Low	Some concerns
Thiruvengadam et al., 2024 [42]	Low	Some concerns	Low	Low	Low	Some concerns
Wang et al., 2020 (CADe-DB) [43]	Low	Low	Low	Low	Low	Low
Nakashima et al., 2023 [44]	Low	Some concerns	Low	Low	Low	Some concerns
Ahmad et al., 2023 [45]	Low	Some concerns	Low	Low	Low	Some concerns
Wei et al., 2023 [46]	Low	Some concerns	Low	Low	Low	Some concerns
Maas et al., 2024 [47]	Low	Some concerns	Some concerns	Low	Low	Some concerns
Wang et al., 2020 (tandem RCT) [48]	Low	Some concerns	Low	Low	Low	Some concerns
Kamba et al., 2021 [49]	Low	Some concerns	Low	Low	Low	Some concerns
Glissen Brown et al., 2022 [50]	Low	Some concerns	Low	Low	Low	Some concerns
Lui et al., 2023 [51]	Low	Some concerns	Low	Low	Low	Some concerns
Shaukat et al., 2022 [52]	Low	Some concerns	Low	Low	Low	Some concerns
Yao et al., 2022 [53]	Low	Some concerns	Low	Low	Low	Some concerns
Jeon et al., 2026 [54]	Low	Some concerns	Low	Low	Low	Some concerns

Abbreviations: CADe, computer-aided detection; RoB 2, Risk of Bias 2. RoB 2 domains: D1, bias arising from the randomization process; D2, bias due to deviations from intended interventions; D3, bias due to missing outcome data; D4, bias in measurement of the outcome; D5, bias in selection of the reported result. Interpretative note: The most frequent source of concern was Domain 2 because masking of the operating endoscopist is inherently difficult in real-time CADe trials: the endoscopist generally knows whether AI assistance is active, potentially influencing inspection behavior, withdrawal technique, or attention. The Wang et al. CADe-DB trial [41] specifically addressed this limitation through a sham-controlled, double-blind design. Histopathological confirmation of detected lesions and the relatively objective nature of the

principal detection outcomes generally reduced concerns regarding outcome measurement. The overall judgments should therefore be interpreted as reflecting predominantly design-related concerns inherent to procedural AI trials rather than evidence of major methodological deficiencies. Judgment categories: Low = low risk of bias; Some concerns = some concerns regarding risk of bias; High = high risk of bias.

Table S2. Funding sources, material support, and author-disclosed conflicts of interest in randomized controlled trials of computer-aided detection (CADe)-assisted colonoscopy summarized in Table 1.

Study	CADe system	Funding and/or material support	Author-disclosed conflicts of interest / industry relationships
Repici et al., 2020 [40]	GI Genius (Medtronic)	No-profit study; no funding received or solicited. GI Genius equipment was loaned by Medtronic.	Relevant author relationships with endoscopy/device companies were disclosed in the publication. Equipment support from Medtronic was explicitly reported.
Rondonotti et al., 2022 [41]	CAD EYE (Fujifilm)	No direct study funding was identified in the published disclosure; commercial relationships were disclosed separately.	E. Rondonotti: Fujifilm speaker honoraria/expert group and Medtronic consultancy; S. Paggi and A. Amato: Fujifilm speaker honoraria; F. Radaelli: Fujifilm speaker honoraria and unrelated research grant; C. Hassan: Fujifilm equipment loan; A. Repici: consultancy/research relationships with Fujifilm, Medtronic, Boston Scientific, Cosmo Pharmaceuticals, and Erbe.
Thiruvengadam et al., 2024 [42]	GI Genius (Medtronic)	GI Genius platform obtained through an American Society for Gastrointestinal Endoscopy (ASGE) Equity Grant.	Authors reported no conflicts of interest

Wang et al., 2020 [43]	EndoScreener (Wision AI)	No funding received. EndoScreener and the sham system were developed by Wision AI and provided free of charge. Company employees were not involved in trial conduct or analysis.	T.M. Berzin reported consulting fees from Shanghai Wision AI during the study; the remaining authors declared no competing interests.
Nakashima et al., 2023 [44]	CAD EYE (Fujifilm)	The article states "There is no funding associated with this study."	H. Nakashima was supported by joint research expenses from Fujifilm; the funder had no role in study design, conduct, or analysis. Other authors reported no COI.
Ahmad et al., 2023 [45]	GI Genius (Medtronic)	No specific commercial funding of the trial was reported in the article.	A. Ahmad: Olympus research funding/equipment loan; N. Suzuki: Olympus consultancy; S. Thomas-Gibson: Olympus speaker fees; B.P. Saunders: consultancy, speaker fees, research funding/equipment loan from Olympus and speaker fees/equipment loan from Fujifilm; remaining authors declared no COI.
Wei et al., 2023 [46]	EndoVigilant	No direct industry study funding was reported in the publication; EndoVigilant was the commercially developed CADe platform evaluated.	Commercial/author relationships were disclosed in the original publication; no direct industry funding of the trial was identified.
Maas et al., 2024 [47]	DISCOVERY (PENTAX Medical)	PENTAX Medical reported as the funding source.	T. Rath: speaker fees including PENTAX, Medtronic and Olympus; C. Spada: Medtronic consultancy and Olympus/PENTAX speaker fees; N. Forbes: PENTAX consultancy, speaker fees and research funding; G. Vanbiervliet: industry

			consultancy/lecture fees including PENTAX and Fujifilm; P. Siersema: research support/consultancy including Magentiq Eye; several other authors declared no COI.
Wang et al., 2020 [48]	EndoScreener (Wision AI)	EndoScreener was provided free of charge by Shanghai Wision AI; company employees were not involved in study design, analysis, or manuscript preparation.	Authors disclosed no conflicts of interest.
Kamba et al., 2021 [49]	Modified YOLOv3-based CADe (LPIXEL/Jikei University)	CADe system developed jointly with LPIXEL Inc.; three coauthors were affiliated with LPIXEL. No separate external financial study sponsor was identified in the publication.	A. Fukuda, A. Tonouchi, and Y. Shimahara were employees/affiliates of LPIXEL Inc.; this commercial affiliation is relevant to interpretation of the study.
Glissen Brown et al., 2022 [50]	EndoScreener (Wision AI)	Investigator-initiated study; research software and study funding provided by Wision LLC. L. Liu additionally received Chinese governmental/public research-grant support.	S. Gross: Olympus consultant; T.M. Berzin: consultant for Wision AI, Fujifilm, and Medtronic; remaining authors reported no conflicts.
Lui et al., 2023 [51]	CADe system developed by NISI (HK) Ltd.	CADe system supplied/developed in association with NISI (HK) Limited; commercial involvement was disclosed.	C.K. Yeung: founder of NISI (HK) Limited; W.K. Leung: advisory committee member for NISI (HK) Limited and consultant for Medtronic; all other authors disclosed no financial relationships.
Shaukat et al., 2022 [52]	SKOUT (Iterative Scopes)	Study funded by Iterative Scopes, Inc.	A. Shaukat, D. Lichtenstein, S.C. Somers, D.C. Chung, and T.R. Church reported research

			funding and/or advisory/consulting relationships with Iterative Scopes; M. Gopal, D.R. Colucci, and S.A. Phillips were Iterative Scopes employees holding stock/stock options; additional relationships were disclosed by individual investigators.
Yao et al., 2022 [53]	ENDOANGEL CADe + CAQ	Academic/institutional development of ENDOANGEL; no commercial sponsor was identified in the published report.	Authors declared no conflicts of interest.
Jeon et al., 2026 [54]	ENADDET-01 real-time CADe	Supported by the Korea Medical Device Development Fund, funded by the Korean government (Project RS-2023-00228946).	No potential conflict of interest relevant to the article was reported.

Abbreviations: ASGE, American Society for Gastrointestinal Endoscopy; CADe, computer-aided detection; CAQ, computer-aided quality improvement; COI, conflict of interest. Note: Funding and conflict-of-interest information was extracted from the funding statements, acknowledgments, author affiliations, and conflict-of-interest disclosures of the corresponding primary publications. Commercial manufacture or development of the evaluated CADe system was not, by itself, classified as commercial study sponsorship. Provision or loan of equipment/software, direct study funding, author employment or company affiliation, consultancy, honoraria, research support, and ownership interests were distinguished where reported. A few of the entries deserve highlighting because they demonstrate why the reviewer's request is useful. Repici explicitly described the trial as no-profit with no funding other than Medtronic's equipment loan. Wang's CADe-DB study similarly reported no funding, although Wision provided the active and sham systems free of charge and Berzin disclosed consulting fees from Wision. In contrast, CADeT-CS received both software and study funding from Wision, the SKOUT trial was funded by Iterative Scopes and included company employees with equity interests, and the DISCOVERY trial reports PENTAX Medical as its funding source. The Nakashima situation is particularly worth wording carefully: the paper states that the study itself had no associated funding, while separately disclosing that Nakashima was supported by joint research expenses from Fujifilm and that Fujifilm had no role in study design, conduct, or analysis. That is why I have preserved this distinction rather than simply labeling the study "Fujifilm-funded." Similarly, the 2026 Jeon trial is cleanly documented as supported by the government-funded Korea Medical Device Development Fund and reports no relevant conflicts.
